# Resting T cells are hypersensitive to DNA damage due to defective DNA repair pathway

**DOI:** 10.1038/s41419-018-0649-z

**Published:** 2018-05-31

**Authors:** Qian Hu, Yujie Xie, Yuanlong Ge, Xin Nie, Jun Tao, Yong Zhao

**Affiliations:** 1 Key Laboratory of Gene Engineering of the Ministry of Education, School of Life Sciences, Sun Yat-sen University, 510006 Guangzhou, People’s Republic of China; 2 Key Laboratory on Assisted Circulation, Ministry of Health, Department of Hypertension and Vascular Disease, The First Affiliated Hospital, Sun Yat-Sen University, 410080 Guangzhou, People’s Republic of China

## Abstract

Blood cells are challenged by intrinsic and exogenous stress that may result in many types of damage to DNA. As a major participant in cell-mediated immunity in blood, T lymphocytes are maintained in their quiescent (resting) state for most of their lives and switch to the proliferating state once stimulated. How resting and stimulated T cells address DNA damage remains largely unknown. Here, we report that while different types of DNA damage are efficiently repaired in stimulated T cells, they result in massive apoptosis of resting T cells. Mechanistically, DNA damage in resting T cells activates the ATM/ATR/DNA-PKcs signaling pathway but fails to induce the formation of γH_2_AX and 53BP1 foci, leading to unrepaired DNA damage that activates apoptosis in a p53-independent but JNK/p73-dependent manner. Mice challenged with high DNA damage stress display far fewer T cells in peripheral blood, lymph nodes, and spleens. Collectively, these results reveal that resting T cells are hypersensitive to DNA damage due to defects in DNA damage repair mechanisms. These findings provide new insight into T-cell function and maintenance of immunity under highly stressed conditions.

## Introduction

Each human cell is challenged by over 10^5^ DNA lesions that come from the environment and cellular metabolism every day^1^. Human cells are equipped with DNA damage repair (DDR) machinery to address a variety of lesions^2^. DNA damage is first detected by ATM, ATR, which stimulate a DDR cascade. Then, various downstream proteins including CHK1, CHK2, and p53 are activated, leading to transient cell cycle arrest that provides time for DNA repair^3^. Meanwhile, Ser139 on H_2_AX is phosphorylated surrounding the damage site, forming a dock to recruit DDR-related proteins^4^. Unrepaired DNA damage induces permanent cell cycle arrest (senescence) or apoptosis, in which p53 has a critical role to balance cell survival and death by transcriptional regulation of both pro-survival and pro-death factors^3^.

Irradiation and chemotherapy agents are used to kill cancer cells by introducing mass DNA damage^5^. This is based on the widely accepted concept that non-proliferating cells are more resistant to IR than proliferating cells^6^. However, it has been reported that the spleen and thymus in which lymphocytes are non-proliferating cells, are highly radiosensitive^7^. The underlying mechanism is unknown. T cells are major lymphocytes that are quiescent most of the time and switch to the proliferating state once stimulated by an antigen. Whether quiescent and stimulated T cells can efficiently repair DNA damage remains to be clarified.

Here, single-stranded and double-stranded breaks were induced in resting or anti-CD3/CD28 stimulated CD4+ T cells. Unexpectedly, we observed that unlike stimulated T cells that rapidly repair DNA damage, resting T cells undergo apoptosis. We discovered that DNA damage responses are defective in resting CD4+ T cells, leading to an incomplete repair of DNA damage. Hypersensitivity of T cells to DNA damage was also observed in the mouse model. The possible reasons for these findings were discussed.

## Results

### DNA damage induces apoptosis in resting T cells

**Fig. 1:**
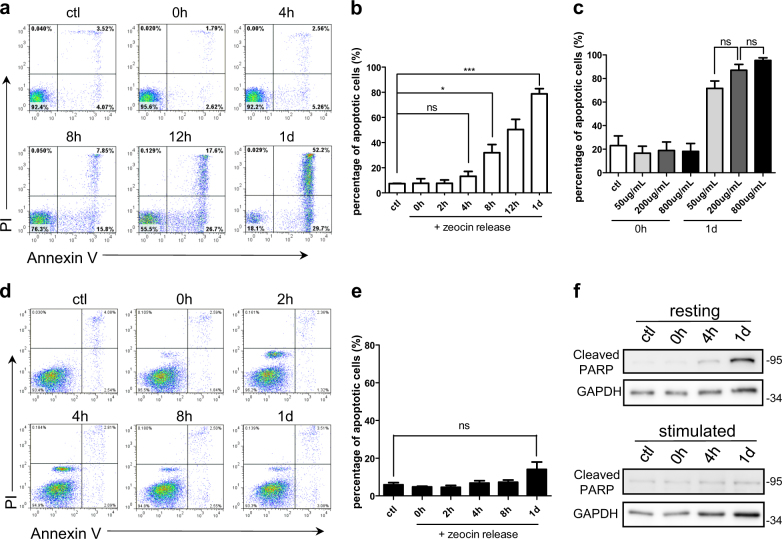
DNA damage induces apoptosis in resting T cells. **a** Freshly isolated (resting) human CD4+ T cells were treated with 200 μg/ml zeocin for 1 h, then released for the indicated time and stained with PI and Annexin V-FITC. The percentage of apoptotic cells were then analyzed by flow cytometry. Ctl indicates fresh CD4+ T cells without zeocin treatment. **b** Quantitation of the percentage of apoptotic (Annexin V positive) cells in **a**. **c** Freshly isolated human CD4+ T cells were treated with low (50 μg/ml), medium (200 μg/ml), and high (800 μg/ml) dose of zeocin and released for one day. Quantitation of flow cytometry was used to determine the percentage of apoptotic (Annexin V positive) cells. **d** Freshly isolated human CD4+ T cells were stimulated with activation beads for two days before treatment with 200 μg/ml zeocin for 1 h. The percentage of apoptotic cells from each time points after zeocin treatment were then analyzed by flow cytometry. Ctl indicates stimulated T cells without zeocin treatment. **e** Quantitation of the percentage of apoptotic (Annexin V positive) cells in **d**. **f** The levels of cleaved PARP in resting and stimulated T cells were determined by western blot. All values are the average ± SEM of three independent experiments. The unpaired student’s two-tailed *t*-test was used to determine the statistical significance (*P < 0.05, ***P < 0.001)

Zeocin, an antibiotic in the bleomycin family, is widely used as an inducer of DNA double-stranded break (DSB)^8,9^. To investigate DDR in human T cell, freshly isolated resting CD4+ T cells or CD4+ T cells stimulated by anti-CD3/CD28-conjugated beads were treated with 200 μg/ml zeocin for 1 h. After release from the zeocin treatment, the percentage of apoptotic resting T cells gradually increased. After one day, 80% of resting T cells underwent apoptosis (Fig. 1a, b). As a control, PBS-treated resting T cells displayed no increase of apoptotic cells (Supplementary Figure 1). To exclude the possibility that a mass of apoptosis is caused by the high dose (200 μg/ml) of zeocin, resting T cells were treated with a much lower dose (50 μg/ml) or a much higher dose (800 μg/ml) of zeocin. We observed that there is no significant difference in the percentage of apoptotic cells between treatments with different doses (Fig. 1c), demonstrating that resting T cells are hypersensitive to DSBs. In contrast, the CD4+ T cells stimulated with anti-CD3/CD28 beads did not undergo apoptosis after the zeocin treatment (Fig. 1d, e). Cell apoptosis were further confirmed by the increased level of cleaved PARP, which was specifically observed in zeocin-treated resting T cells (Fig. 1f).

Since DSB is highly toxic compared to other kinds of DNA lesions, we then tested the apoptosis of resting T cells when treated with H_2_O_2_ or ionizing radiation (X-ray), which induce single-stranded breaks (SSBs) or a mixture of DSBs and SSBs, respectively^2^. Like zeocin, both X-ray (Supplementary Figure 2a–d) and H_2_O_2_ (Supplementary Figure 2e) led to apoptosis of resting T cells. In contrast, stimulated T cells displayed no apoptosis upon treatments.

### DNA damage was not efficiently repaired in resting T cells

**Fig. 2:**
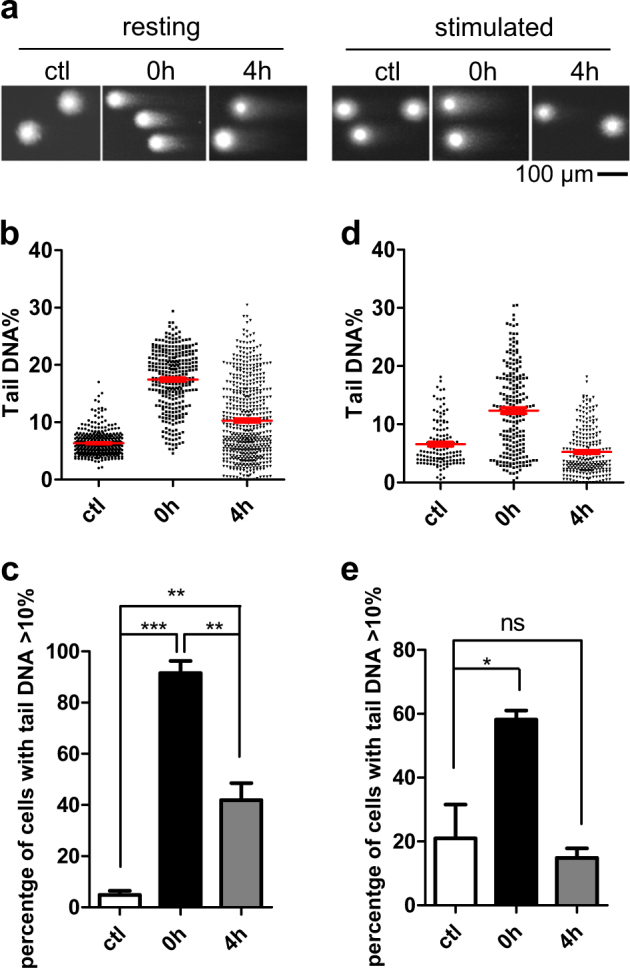
DSB was not sufficiently repaired in resting T cells. **a** Representative results of neutral comet assay. Resting or stimulated CD4+ T cells were treated with 200 μg/ml zeocin for 1 h then released at the indicated times. **b**, **c** The percentages of DNA in the tail for each resting CD4+ T cell (**b**) and the percentage of resting cells with over 10% tail DNA (**c**) were measured. Cells were isolated from three healthy donors, including control (*n* = 373), 0 h after zeocin treatment (*n* = 308) and 4 h after zeocin treatment (*n* = 547). **d**, **e** Percentage of DNA in the tail for each stimulated CD4+ T cell (**d**) and the percentage of stimulated cells with over 10% tail DNA (**e**) were measured. Cells were isolated from three healthy donors, including the control (*n* = 125), 0 h after zeocin treatment (*n* = 219) and 4 h after zeocin treatment (*n* = 242). All values are the average ± SEM of three independent experiments. The unpaired student’s two-tailed *t*-test was used to determine the statistical significance (*P < 0.05, **P < 0.01, ***P < 0.001)

Unrepaired DNA damage may result in apoptosis. To investigate the repair of DNA damage in resting and stimulated T cells, neutral and alkaline comet assays were performed to detect only DSBs and multiple DNA lesions (DSBs, SSBs, and alkali-labile sites), respectively^10^. As expected, both zeocin and H_2_O_2_ induced a significant amount of DNA damage, leading to DNA fragments that leave the genome and form “tail” during the comet assay. A percentage of tail DNA was used to indicate the abundance of fragments induced by DNA lesions. To exclude the interference from nuclear condensation and DNA fragmentation caused by apoptosis, cells released from treatment for 4 h which showed no significant apoptosis (Fig. 1b), were assayed. The results showed that although a significant number of DSBs were repaired in 4 h after the zeocin treatment in resting T cells, the average level of DSBs was still higher than untreated cells (Fig. 2b). Accordingly, more than 40% of cells displayed higher DSBs levels than baseline (10% tail DNA signal) (Fig. 2c). As with the zeocin treatment, resting CD4+ T cells failed to completely repair H_2_O_2_-induced DNA lesions (Supplementary Figure 3). It is worth noting that although average level of DNA lesions in cells decreased 4 h after treatment (Supplementary Figure 3b), the percentage of cells with higher level of DNA lesions than the baseline did not decrease significantly, suggesting incomplete repair of DNA lesions in resting CD4+ T cells (Supplementary Figure 3c). In contrast, the DNA damage (zeocin-induced DSBs or H_2_O_2_-induced DNA lesions) in stimulated T cells was rapidly repaired as they returned to the baseline level in 4 h after treatment (Fig. 2a, d, e; Supplementary Figure 3a, d, e). Collectively, these results demonstrate that resting CD4+ T cells are unable to efficiently repair DNA damage over the course of 4 h especially for H_2_O_2_-induced DNA lesions, which may induce cell apoptosis.

### DDR machinery is deficient in resting T cells

**Fig. 3:**
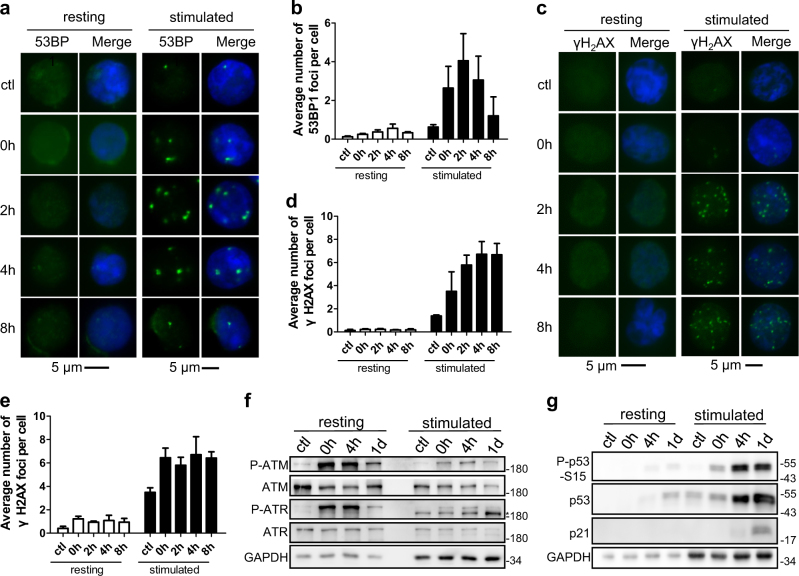
DDR machinery is deficient in resting T cells. **a** Resting and stimulated CD4+ T cells were treated with 200 μg/ml zeocin for 1 h and release for indicated times. Untreated (non-damaged) resting and stimulated T cells were used as negative control (ctl). 53BP1 foci were analyzed by immunofluorescence with 53BP1 antibody. Cell nuclei were stained with DAPI as shown in the “merge” images. **b** Quantitation of the data in **a**. The average number of 53BP1 foci per cell was determined. **c** Resting and stimulated CD4+ T cells were treated with 200 μg/ml zeocin for 1 h and released at the indicated times. Untreated (non-damaged) resting and stimulated T cells were used as negative control (ctl). γH2AX foci were analyzed by immunofluorescence with γH2AX antibody. Cell nuclei were stained with DAPI as shown in the “merge” images. **d** Quantitation of the data in **c**. The average number of γH_2_AX foci per cell was determined. **e** Resting and stimulated CD4+ T cells were treated with 25 μM H_2_O_2_ for 10 min then released at indicated times. γH2AX foci were analyzed by immunofluorescence with γH2AX antibody. The average number of γH_2_AX foci per cell was determined. **f**, **g** Resting and stimulated CD4+ T cells from each time point post zeocin treatment were analyzed by western blot. The asterisk indicates an unspecific band. All values are the average ± SEM of three independent experiments

It has been reported that the activation of T cells is accompanied by a comprehensive change of gene expression and chromatin structure^11–13^. We suspected that the DDR machinery may be different between resting and stimulated T cells, leading to different capacities for DDR. In support of this theory, we found that 53BP1 foci, which is an indicator for the activation of DDR, were frequently observed in stimulated CD4+ T cells (~4 foci/cell). However, barely detectable 53BP1 foci were observed in resting CD4+ cells (Fig. 3a, b). We also examined γH_2_AX foci, the earliest marker of DDR that provides a docking site for other DDR factors^2^. Strikingly, γH_2_AX foci were also deficient in zeocin-treated resting cells (Fig. 3c, d). Similar results were obtained in resting T cells treated with H_2_O_2_ (Fig. 3e). We also examined the formation of γH_2_AX or 53BP1 foci in resting and stimulated T cells irradiated with X-ray (Supplementary Figure 4). Similar to previous report that γH_2_AX or 53BP1 foci were detectable in irradiated resting T cells^14,15^. However, the average number of foci in resting T cell is much lower than that in stimulated T cell, demonstrating that the activation of DDR in resting CD4+ T cells is largely suppressed. Consistently, we observed that transcriptional levels of DDR downstream proteins in zeocin-treated resting T cells, including CHK1, CHK2 and many factors in non-homologous end joining (NHEJ), homologous recombination (HR) and Fanconi Anaemia pathway, were much lower than that in zeocin-treated stimulated T cells (Supplementary Figure 5).

ATM, ATR, and DNA-PKcs are kinases that phosphorylate H_2_AX when DNA damage occurs^2^. We found that all these kinases were activated by phosphorylation in response to zeocin treatment in both resting and stimulated CD4+ T cells (Fig. 3f; Supplementary Figure 6a). Consistently, we detected a significant amount of γH_2_AX by western blot after 4 h of treatment in resting T cells (Supplementary Figure 6b). Thus, it appears that activated ATM, ATR, and DNA-PKcs phosphorylated H_2_AX. These γH_2_AX are diffused instead of locating surrounding the DNA damage site, leading to the suppression of DDR. Moreover, we also found that p53 is barely activated by the phosphorylation at Ser15 and its target protein p21 is undetectable in zeocin-treated resting T cells. In contrast, p53 is rapidly activated in stimulated T cells (Fig. 3g).

### DDR machinery is efficient in quiescent fibroblasts

**Fig. 4:**
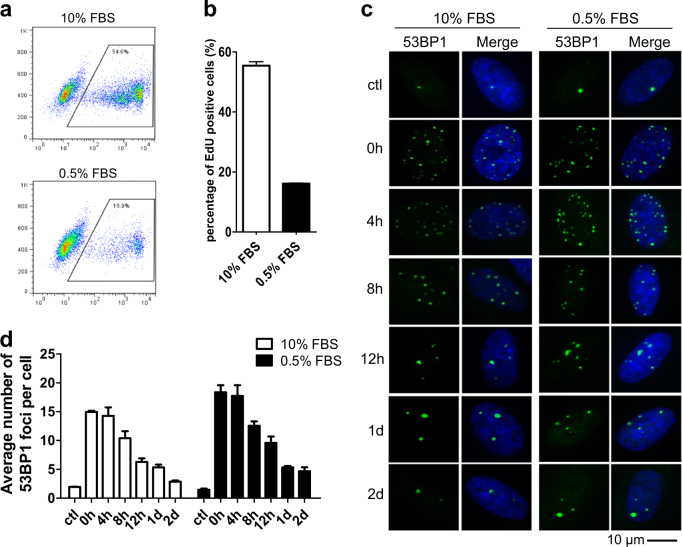
DDR machinery is efficient in quiescent fibroblasts. **a** MRC5 fibroblast cells were starved by culturing with medium containing 0.5% FBS or 10% FBS for 24 h and then labeled with EdU for 12 h. The incorporation of EdU was then determined by flow cytometry. **b** Quantitation of the data in **a**. The percentage of EdU-positive cells were determined. **c** 53BP1 foci in quiescent (0.5% FBS) and proliferating (10% FBS) MRC5 cells were analyzed by immunofluorescence with 53BP1 antibody. Cell nuclei were stained with DAPI as shown in the “merge” images. **d** Quantitation of the 53BP1 foci in **c**. The average number of 53BP1 foci per cell was determined. All values are the average ± SEM of three independent experiments

Resting CD4+ T cells are quiescent. Once stimulated, these resting T cells proliferate and generate a large clone of antigen-specific cells. We thus suspected whether the deficiency of DDR in resting T cells was caused by its quiescent state. To test this, MRC5 fibroblast cells were subjected to serum starvation (0.5% FBS) for 24 h. Their proliferation state was determined by flow cytometry after 12 h labeling with EdU. The vast majority of MRC5 fibroblast cells were EdU negative, demonstrating their quiescent state (Fig. 4a, b). Strikingly, DDR is activated normally upon zeocin treatment in these cells as it is in proliferating cells (Fig. 4c). 53BP1 foci decreased gradually and returned to background level after two days (Fig. 4d). Therefore, the deficiency in DDR is not due to the quiescent state of cells, but is a specific characteristic of resting T cells.

### DSBs induce T cells apoptosis through p53-independent pathway

**Fig. 5:**
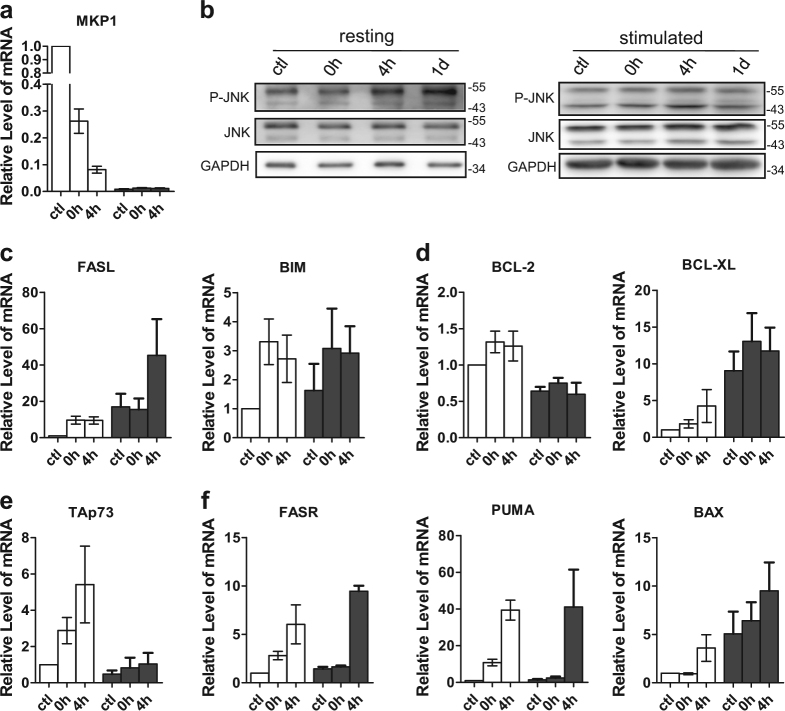
DSBs induce resting T cells apoptosis through p53-independent way. Resting and stimulated CD4+ T cells from each time point post zeocin treatment were analyzed by western blot or Q-PCR. Ctl means T cells without zeocin treatment. **a** Q-PCR analysis of MKP1. **b** Western blot analysis of phosphorylated JNK, total JNK and GAPDH. The upper band of phosphorylated or total JNK represents JNK2 and JNK3. The lower band of phosphorylated or total JNK represents JNK1. **c** Q-PCR analysis of FasL and BIM. **d** Q-PCR analysis of BCL-2 and BCL-XL. **e** Q-PCR analysis of TAp73. **f** Q-PCR analysis of FASR, PUMA, and BAX. All the RT-PCR data were normalized to the mRNA level of CD3E. All values are the average ± SEM of three independent experiments

While the activated p53 promotes cell cycle arrest and DNA repair, it also drives the intrinsic and extrinsic apoptosis pathway by the upregulation of pro-apoptosis factors^3^. Since p53 was not fully activated in resting CD4+ T cells (Fig. 3g), we speculated that p53-independent pathways might be activated by DNA damage in resting CD4+ T cells that promotes apoptosis. Indeed, when resting T cells from p53-knockout mice were treated with zeocin or H_2_O_2_, significant increase of apoptotic cells were observed (Supplementary Figure 7a), demonstrating the existence of p53-independent pathway leading to T-cell apoptosis. Consistently, in human resting T cells, the transcriptional levels of NOXA and PTEN, which are downstream genes of p53, were not changed after zeocin treatment (Supplementary Figure 7b). Instead, we observed that the level of MKP1, which negatively regulates the activation of JNK^16^, decreased (Fig. 5a). Accordingly, phosphorylated/activated JNK increased post zeocin treatment (Fig. 5b), resulting in upregulation of its transactivation targets FASL and BIM (Fig. 5c)^17^. JNK has been reported to be involved in the apoptosis of T lymphocytes^18^. In consistent with previous report^19^, we observed the activation of JNK pathway (MKP1, FASL, and BIM) in stimulated T cells regardless of zeocin treatment (Fig. 5a–c). Meanwhile, highly expressed BCL-XL was detected (Fig. 5d), which may act to suppress the apoptosis of stimulated T cells^20^.

Additionally, p73 is a structural and functional homolog of p53^21^. Its transcriptionally active isoform (TAp73) has been reported to be activated by DNA damage, which then induces apoptosis through the transactivation of FasR (CD95), PUMA and BAX^22–26^. We found that the abundance of TAp73 mRNA is significantly increased in resting CD4+ T cells treated with zeocin (Fig. 5e). As expected, the expression of FasR, PUMA and BAX are upregulated in response to the zeocin treatment (Fig. 5f). Moreover, the level of pro-survival factor BCL-2 displayed no obvious change (Fig. 5d). In contrast, in stimulated T cells p73 was not activated (Fig. 5e).

### Defective T cell pools in zeocin-treated mice

**Fig. 6:**
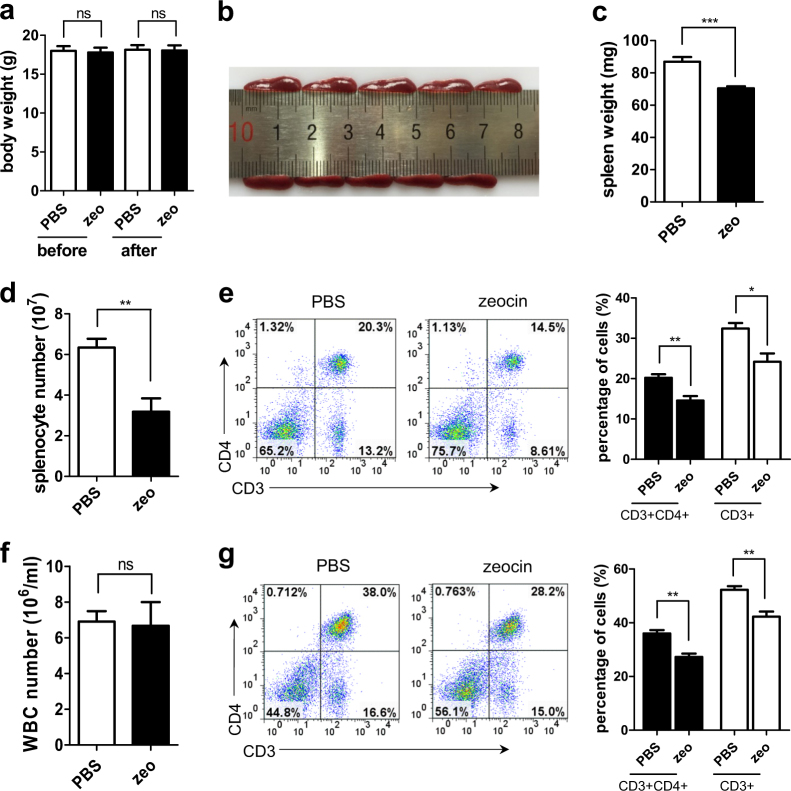
Defective T cell pool in zeocin-treated mice. Six-week-old female BALB/c mice were injected with PBS or zeocin (320 mg/kg) and killed 3 days later. **a** The body weight of mice before and after PBS and zeocin treatment were measured. **b** Representative image of spleens from mice treated with PBS (upper) and zeocin (bottom) are shown. **c** The weight of spleens from mice treated with PBS or zeocin was measured. **d** The total cell number of splenocytes in the spleens of mice treated with PBS or zeocin were determined. **e** Left: representative results of flow cytometry that show CD3 and CD4 staining of splenocytes. Right: Quantitation of the proportion of T cell or CD4+ T cell in splenocytes. **f** The concentration of white blood cells in peripheral blood was counted. **g** Left: representative results of flow cytometry that show CD3 and CD4 staining of white blood cells. Right: Quantitation of the proportion of T cell and CD4+ T cell in white blood cells. Data are representative of five to six mice for each group. All values are the average ± SEM. The unpaired student’s two-tailed *t*-test was used to determine the statistical significance (*P < 0.05, **P < 0.01, ***P < 0.001)

We next evaluated the effect of DNA damage on T cells pools in vivo. BALB/c mice were killed 3 days after being injected with zeocin or PBS. We found the spleens of zeocin-treated mice were smaller and lighter than those of the control mice (Fig. 6b, c), with no significant changes in their body weight (Fig. 6a). The weight of the sampled kidneys and livers were also not affected by the zeocin treatment (Supplementary Figure 8a and b). The number of splenocytes in zeocin-treated mice were only about 50% of those in control mice (Fig. 6d). Moreover, flow cytometry analysis revealed that both the proportion of CD3+ and CD3+CD4+ cells in splenocytes were much lower in zeocin-treated mice (Fig. 6e). In peripheral blood, the percentages of CD3+ and CD3+CD4+ cells were significantly less in zeocin-treated mice despite unchanged total white blood cells counts (Fig. 6f, g). In addition, the immune cell in the lymph nodes (LN) were much lower in zeocin-treated mice compared to the control mice (Supplementary Figure 8c). Since over 80% of the cells in LN were T cells (Supplementary Figure 8d), the CD3+ and CD3+CD4+ cells were much lower in zeocin-treated mice than those in control mice. In summary, these results suggested that the T cell pools in mice were severely impaired by zeocin.

## Discussion

DDR machinery is activated to repair endogenous and exogenous DNA lesions. Our results reveal that when facing DNA damage such as DSBs and SSBs, resting and stimulated CD4+ T cells behave differently. Resting CD4+ T cells, which are deficient in DDR, tend to undergo apoptosis in the p53-independent pathway, whereas stimulated CD4+ T cells rapidly repair DNA damage. These results are consistent with previous finding that quiescent B cells are less efficient than stimulated B cells in repairing DNA damage^27^. Also, it has been reported that HR and Fanconi Anaemia pathway-mediated DDR are significantly decreased in terminally differentiated macrophages^28^. Furthermore, broad attenuation of DNA repair pathways was observed in quiescent hematopoietic stem cells, resulting in accumulation of DNA damage^29^. Together with our finding in T cells, these observations strongly suggest decreased/insufficient capacity for repairing DNA damage in non-proliferating immune cells.

We observed that although ATM and ATR are activated, γH_2_AX and 53BP1 cannot be recruited to the damage sites (Fig. 3), indicating the signal is blocked in the early stage of DDR. It has been described that DDR signaling might be affected by chromatin structure. For instance, the various modification of histones at the DNA damage site is involved in the recruitment of DDR-related factors to damage sites and thus modulates DDR signaling^30,31^. Indeed, it has been reported that the chromatin structure of resting T cells is highly compacted, while the stimulation of T cells results in marked changes in histone modification and relaxation of the chromatin structure^13^. Moreover, it has been demonstrated that gene transcription is widely suppressed in resting T cells, and transcriptional level of many DDR-related genes is low that may not be sufficient to conduct signal transduction, leading to incomplete repair of DNA damage^11,12^. Comparing to the resting T cells, a large number of γH_2_AX/53BP1 foci were formed in response to DNA damage in stimulated T cells. In addition to potential explanations above, another possibility is that rapidly proliferating T cells may convert SSBs or other DNA lesions to DSBs during DNA replication, leading to the activation of DDR.

It has been proposed that low levels of DNA damage activate p53 by primary modifications to induce cell cycle arrest, while severe DNA damage leads to constant activation of p53 by further modifications that initiate apoptosis^3,32^. However, the regulation of p53 and its downstream response to DNA damage is complicated and depends on cell type and stimuli^3^. Our data revealed that the phosphorylation of p53 at Ser15 is very low in resting T cells treated with zeocin (Fig. 3g), in agreement with previous studies that neither an increase of p53 protein level nor its phosphorylation was detected in quiescent PBMCs after irradiation^33^. Moreover, our and other’s data demonstrated that DNA damage induces apoptosis in p53-deficient mice (Supplementary Figure 7a)^34,35^ and human cells^36^, indicating the existence of p53-independent apoptosis. Our results showed that JNK and p73 pathway are activated when facing DNA damage, leading to rapid cell apoptosis. In this context, p53 may not be a major player in preventing the malignant proliferation of T cells, different from its well-known function in suppressing tumorigenesis. Indeed, while p53 is deficient or mutated in more than 60% of human primary tumors, only about 10–20% of hematological cancers show dysfunction of p53^37^.

Our discovery also reveals that quiescent fibroblasts repair DSBs as efficiently as proliferating fibroblasts (Fig. 4c,d), indicating that the deficiency of DDR in resting T cells is not due to the quiescent state of cells. Given that T cells are directly exposed to endogenous and exogenous stress, such as a variety of reagents in blood that may induce DNA damage, hypersensitivity to DNA damage renders T cells highly at risk for apoptosis. Our in vivo experiment also demonstrated that zeocin treatment induces significant decrease in T cells in peripheral blood and immune organs of mice. Considering the large population of T cells in blood, it may be less costly to kill these cells when they are attacked by large amounts of DNA damage. Moreover, hematopoietic stem cells (HSCs) may quickly compensate for the loss of immune cells. In contrast, once stimulated, T cells are programmed to undergo clonal expansion to participate in the immune response. Thus, it is important for the cells to obtain robust repair abilities to enhance immune response and reduce the accumulation of DNA damage which may result in carcinogenesis. However, this unique design may be challenged by a large amount of exogenous DNA damage induced by environment irradiation or cancer therapy (chemotherapy and radiotherapy), leading to severe impairment in the pool of naive T lymphocytes and decrease of their immune capacity. Thus, the decreased immunity due to hypersensitivity of T cells to DNA damages should be considered when human is exposed to high dose of irradiation or patients are treated with chemotherapy or radiotherapy.

## Materials and methods

### Preparation of CD4+ T cells from human peripheral blood

All samples and data were anonymously performed. All the experiments was approved by the review board and ethics committee of Sun Yat-Sen University. PBMCs were isolated from whole blood drew from informed healthy donors with Human Whole Blood Mononuclear Separation Medium (Sangon Biotech, China) or directly purchased from Leidebio, Guangzhou, China. CD4+ T cells were negatively isolated from PBMC by using Dynabeads following the manufacturer’s instructions (ThermoFisher).

### Cell culture and treatments

CD4+ T cells stimulation was performed with Dynabeads Human T-Activator CD3/CD28 (Gibco) for 2 days. Resting and stimulated T cells were cultured in RPMI 1640+GlutaMAX (Gibco) supplemented with 10% FCS (Gibco), penicillin/streptomycin (Gibco) and 30 U/ml recombinant human IL-2 (PeproTech) at 37 °C in a humidified 5% CO_2_ incubator. To induce DSBs, the cells were treated with 200 μg/ml zeocin for 1 h at 37 °C. To induce the mixture of SSBs and DSBs, the cells were treated with 25 μM H_2_O_2_ for 10 min at room temperature.

MRC5 fibroblasts were cultured in DMEM supplemented with 10% FBS or 0.5% FBS (for serum starvation) and penicillin/streptomycinat 37 °C in a humidified 5% CO_2_ incubator. DSBs were induced by treating the cells with 100 μg/ml zeocin for 1 h at 37 °C.

### Mice

Four-week-old female p53−/− C57BL mice were purchased from Beijing Biocytogen Co. Four-week-old female wildtype (wt) C57BL mice were purchased from the Laboratory Animal Center of Sun Yat-sen University. Resting T cells were negatively isolated from spleen of p53−/− or wt mice using Dynabeads following the manufacturer’s instructions (Invitrogen). Six-week-old female BALB/c mice were purchased from the Laboratory Animal Center of Southern Medical University. BALB/c Mice were injected with 100 μl PBS or zeocin (320 mg/kg). Three days later, mice were killed. Cells were isolated from subiliac LN and spleen by mashing through 70 μm sieve. All experimental protocols concerning the handling of mice were approved by the institutional animal care and use committee of Sun Yat-sen University.

### Apoptosis assay and flow cytometry

Cell apoptosis was determined with Annexin V-FITC Apoptosis Detection Kit (KeyGen Biotech, China) according to the manufacturer’s instructions. Apoptotic cells were determined by flow cytometry with FACSAria II (BD).

To detect the proportion of T cells, cells were stained with APC anti-human CD3 (Biolegend), FITC anti-human CD4 (Biolegend), FITC anti-mouce CD3 (Biolegend) or APC anti-mouce CD4 (Biolegend), and detected by flow cytometry (FACSAria II, BD).

### Comet assay

Neutral or alkaline comet assay was used to detect only DSBs and multiple DNA lesions (DSBs, SSBs, and alkali-labile sites), respectively. Briefly, T cells were harvested and mixed with 0.5% low melting temperature agarose and layered on slides pre-coated by 1.5% normal agarose. For the neutral assay, slides were lysed in 2.5 M NaCl, 100 mM EDTA, 10 mM Tris (pH 8.0), 0.5% Triton X-100, 3% DMSO, 1% *N*-lauroylsarcosine overnight at 4 °C and then electrophoresis in 300 mM sodium acetate, 100 mM Tris-HCl, 1% DMSO at 1.5 V/cm for 20 min. For the alkaline assay, lysis buffer was composed of 2.5 M NaCl, 100 mM EDTA, 10 mM Tris (pH 10.0), 1% Triton X-100, and electrophoresis buffer was composed of 300 mM NaOH, 1 mM EDTA. After neutralization with 0.4 M Tris-HCl (pH 7.3), slides were washed and dried with ethanol. The slides were then mounted with Vectashield mounting medium containing DAPI (Vector Laboratories) and visualized under fluorescence microscopy (Axio Observer Z1, ZEISS). Analysis was performed with CASP.

### Immunofluorescence

Immunofluorescence was performed as previously described with a minor modification^38^. Briefly, T cells were washed once with PBS, re-suspended in PBS and planted on poly-l-lysine-treated coverslips. After fixation with 4% paraformaldehyde and permeabilization with Triton, the coverslips were incubated sequentially with primary antibody (anti-53BP1, Novus Biologicals; or anti-γH_2_AX, Cell Signaling Technology) and fluorescent labeled second antibody. Coverslips mounted with Vectashield mounting medium containing DAPI (Vector Laboratories) were visualized with fluorescent microscope.

### Western blotting

Proteins were separated with SDS-PAGE and transferred to PVDF membrane. The following antibodies were incubated with membrane: anti-ATM (Abcam), anti-ATM-pS1981 (Abcam), anti-ATR (Abcam), anti-ATR-pS428 (Abcam), anti-DNA-PKcs-pS2056 (Abcam), anti-p53(Santa Cruz), anti-p21(Santa Cruz), anti-cleaved PARP (Cell Signaling Technology), anti-p53-S15 (Cell Signaling Technology), anti-JNK (Cell Signaling Technology), anti-phosphor-JNK (Cell Signaling Technology), anti-GAPDH (Proteintech), anti-γH_2_AX (Cell Signaling Technology). HRP-conjugated anti-rabbit or anti-mouse (KPL, Inc) were then used.

### RNA isolation and Q-PCR

Total RNA was extract from resting CD4+ T cells with RNAiso Plus (Takara) and cDNA was prepared with PrimeScript RT reagent kit (Takara) following the manufacturer’s instruction. Real-time PCR reaction were prepared with 2×RealStar Power SYBR Mixture (GenStar, China) and analyzed on LightCycler 480 (Roche). CD3E was used for normalization^39^. Q-PCR primers are shown in the Supplementary Table.

### EdU assay

Following serum starvation, MRC5 cells were cultured with medium supplemented with 10 μM EdU for 12 h. Trypsinized cells were washed with PBS and fixed with 70% ethanol overnight at 4 °C. After washing with PBS, cells were stained at room temperature for 30 min with staining buffer (10 μM FAM-azide, 1 mM CuSO_4_ and 10 mM sodium ascorbate in PBS). After washing with PBST for three times, the percentage of EdU-positive cells were detected by flow cytometry with FACSAria II (BD).

### Statistics

GraphPad Prism 5 was used for statistical analysis. Results are shown as means ± SEM and the unpaired student’s two-tailed *t*-test was used to determine the statistical significance (*P < 0.05; **P < 0.01. ***P < 0.001)

## Electronic supplementary material

Supplementary figures

Supplemetary Figure legend

Supplementary table
