# Supplementary material for: Resting T cells are hypersensitive to DNA damage due to defective DNA repair pathway
Source: Cell Death Dis. 2018 May 31;9(6):662. doi: 10.1038/s41419-018-0649-z (PMC5981309; doi:10.1038/s41419-018-0649-z)
Supplement: Supplementary file 1 — Supplementary figures [file 41419_2018_649_MOESM1_ESM.pptx]

## Slide 1
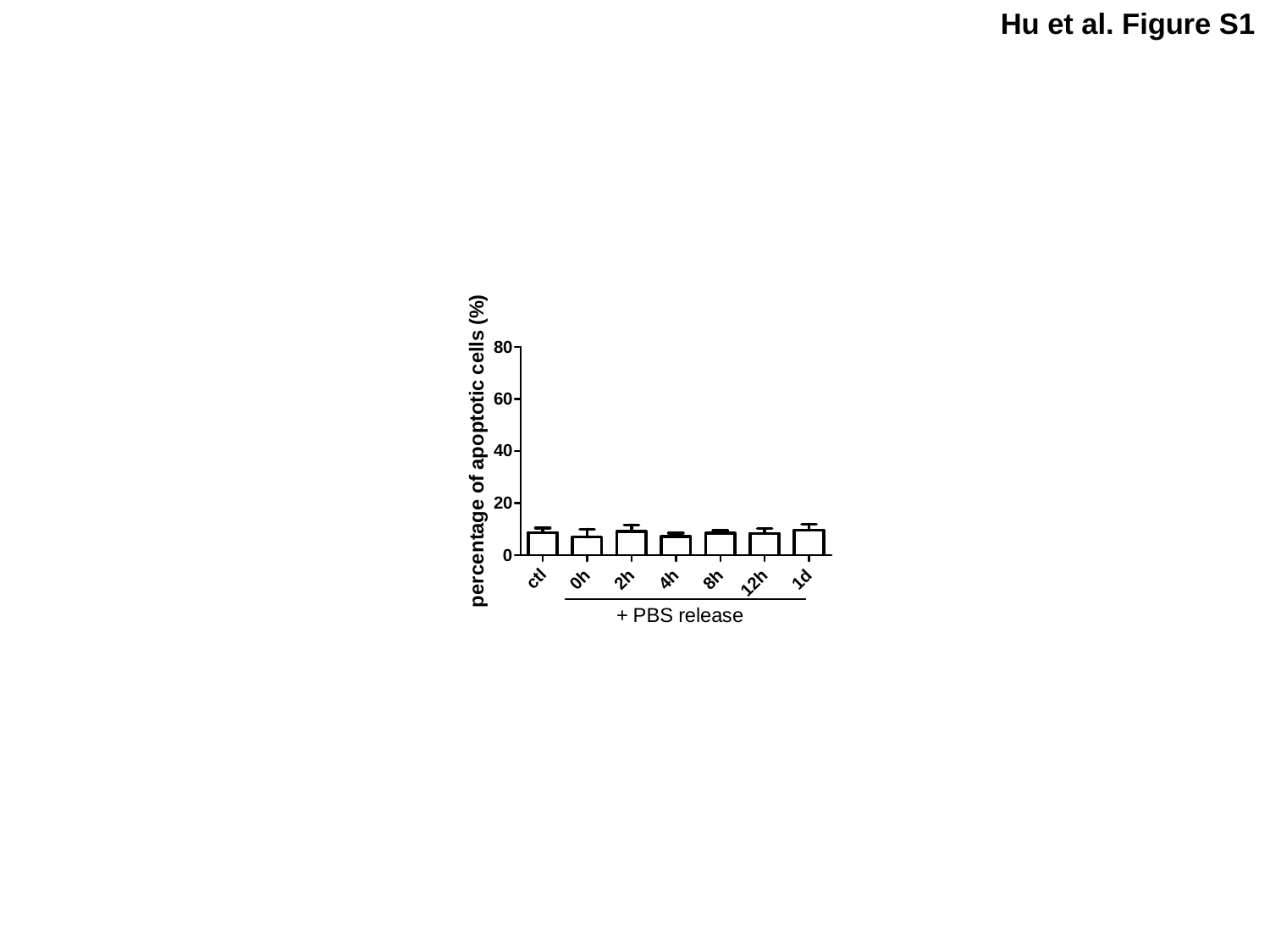

Hu et al. Figure S1

## Slide 2
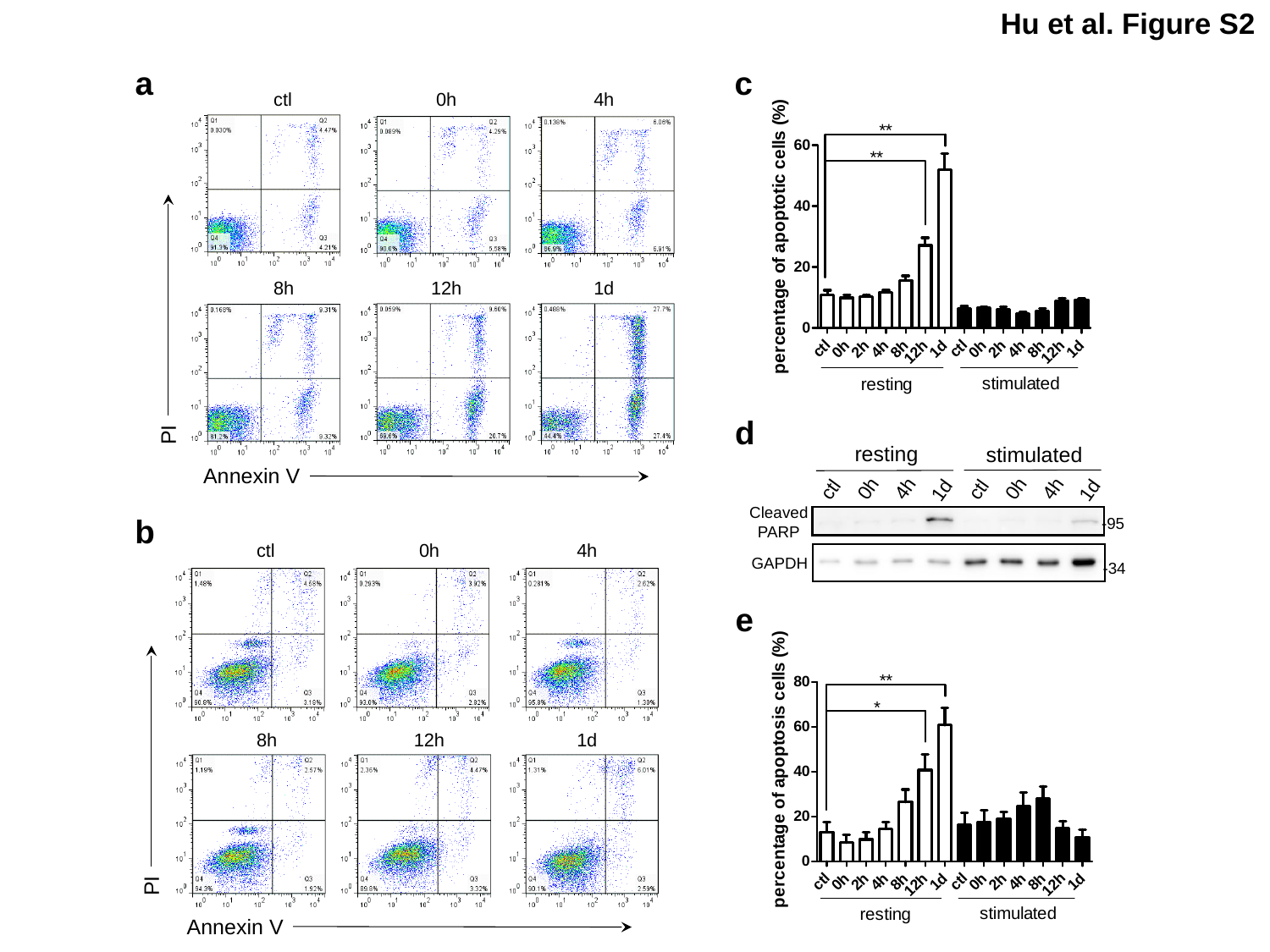

Hu et al. Figure S2
a
c
ctl
0h
4h
8h
12h
1d
PI
Annexin V
d
resting
stimulated
ctl
ctl
0h
4h
0h
4h
1d
1d
Cleaved
PARP
-95
GAPDH
-34
b
ctl
0h
4h
8h
12h
1d
PI
Annexin V
e

## Slide 3
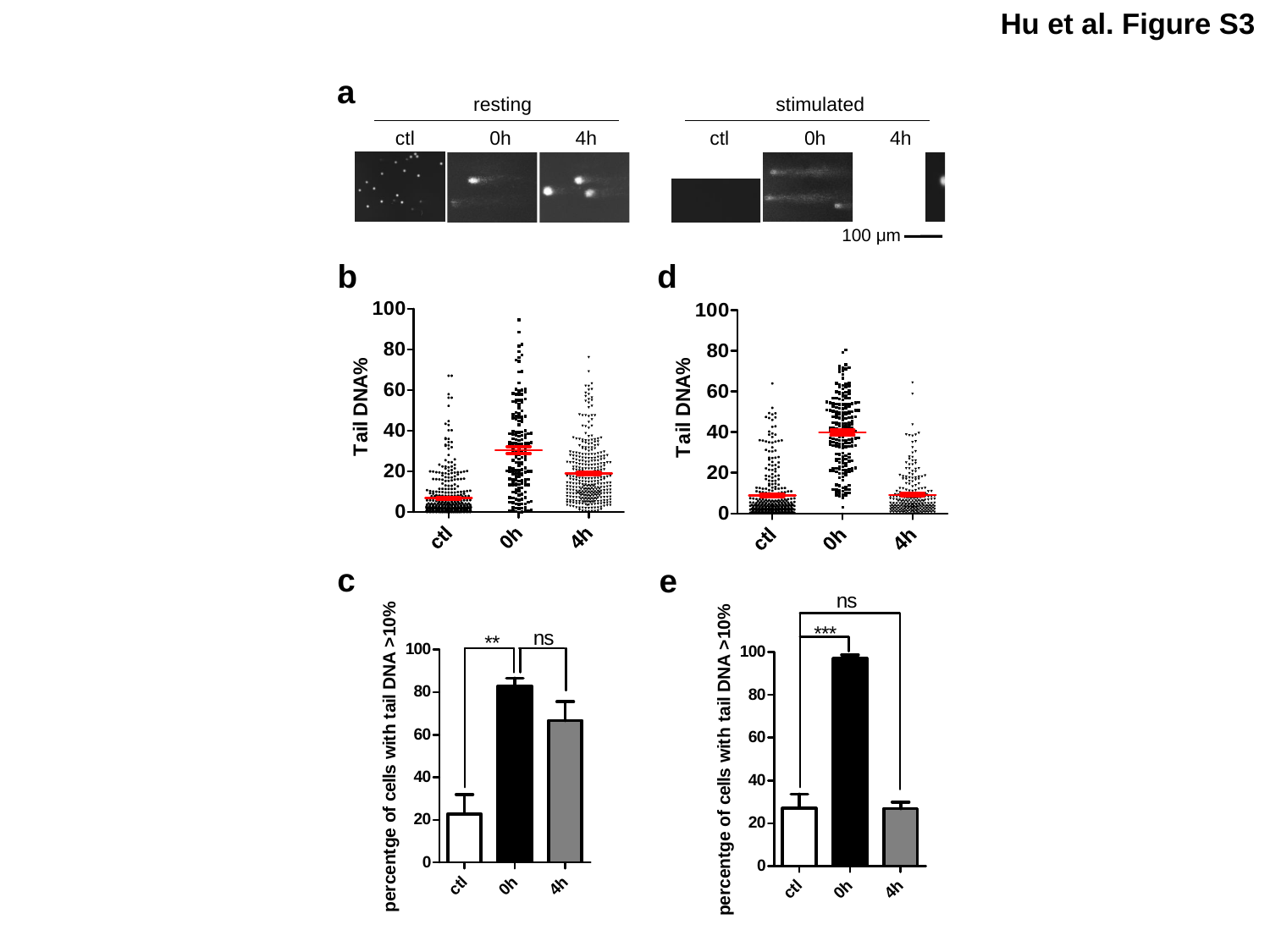

Hu et al. Figure S3
a
stimulated
resting
ctl
0h
4h
ctl
0h
4h
100 μm
b
d
c
e

## Slide 4
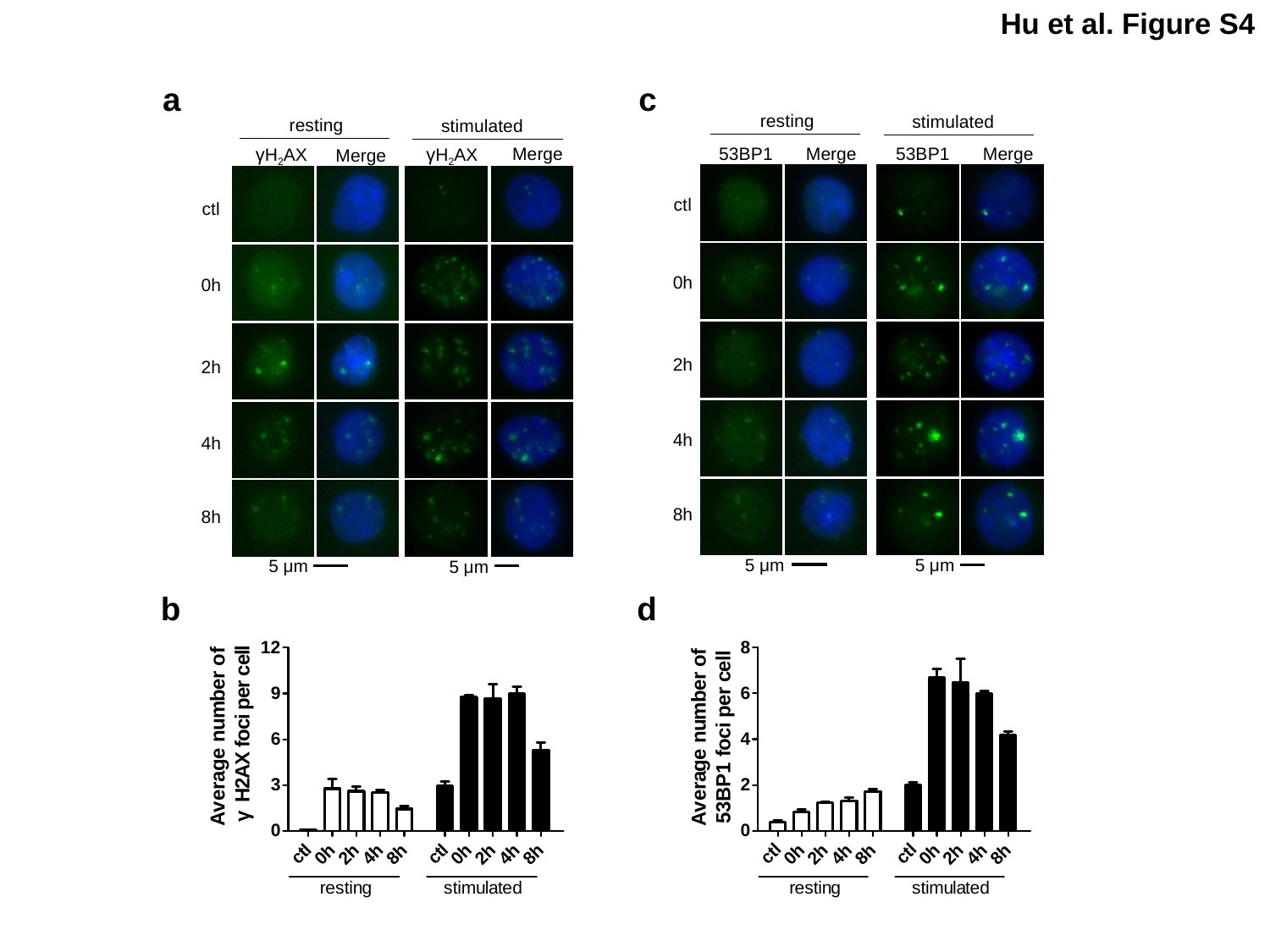

Hu et al. Figure S4
a
c
resting
stimulated
Merge
Merge
53BP1
53BP1
ctl
0h
2h
4h
8h
5 μm
5 μm
resting
stimulated
γH2AX
Merge
γH2AX
Merge
ctl
0h
2h
4h
8h
5 μm
5 μm
b
d

## Slide 5
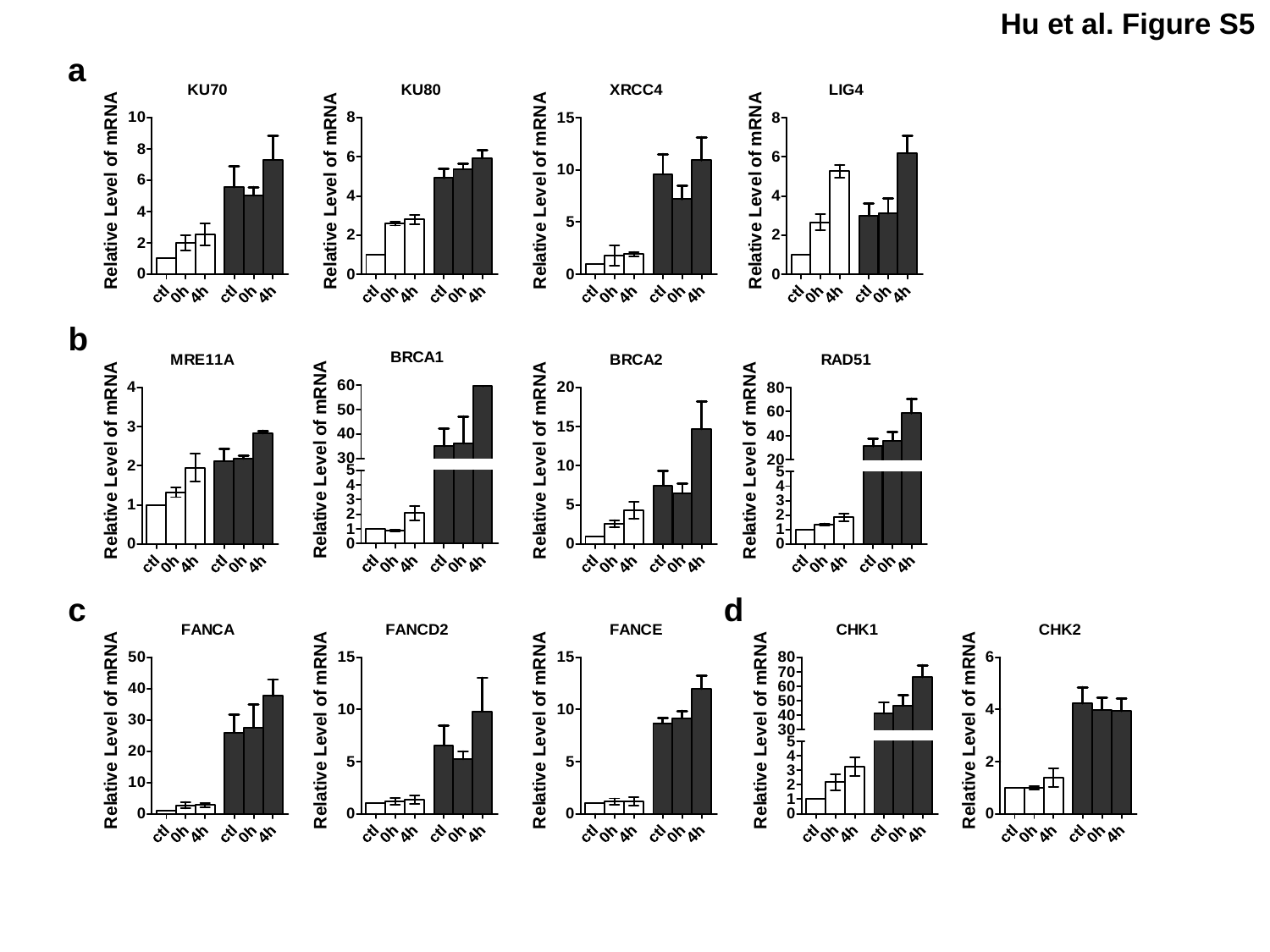

Hu et al. Figure S5
a
b
d
c

## Slide 6
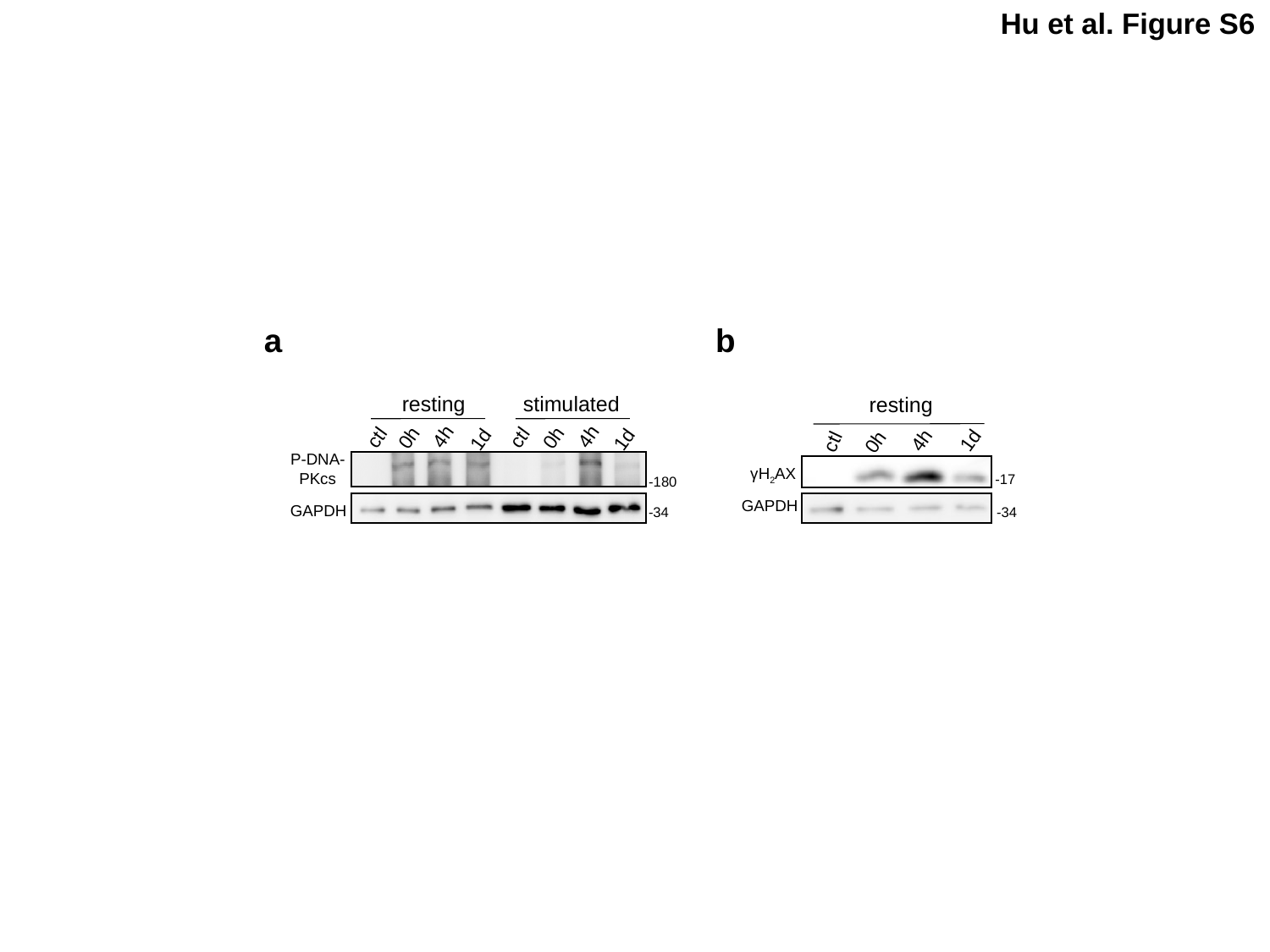

Hu et al. Figure S6
a
b
resting
stimulated
ctl
ctl
4h
4h
0h
0h
1d
1d
P-DNA-PKcs
-180
GAPDH
-34
resting
ctl
4h
1d
0h
γH2AX
-17
GAPDH
-34

## Slide 7
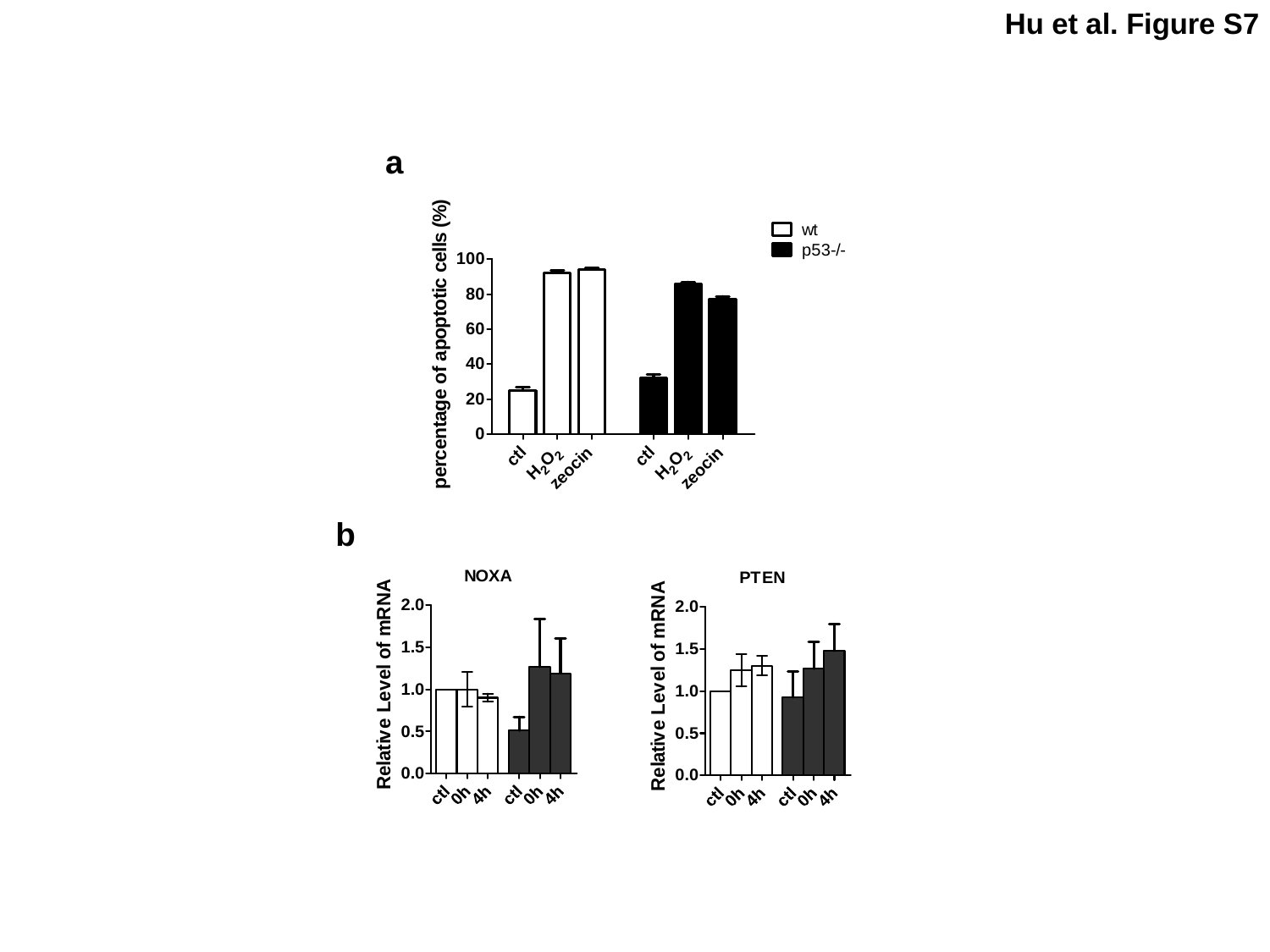

Hu et al. Figure S7
a
b

## Slide 8
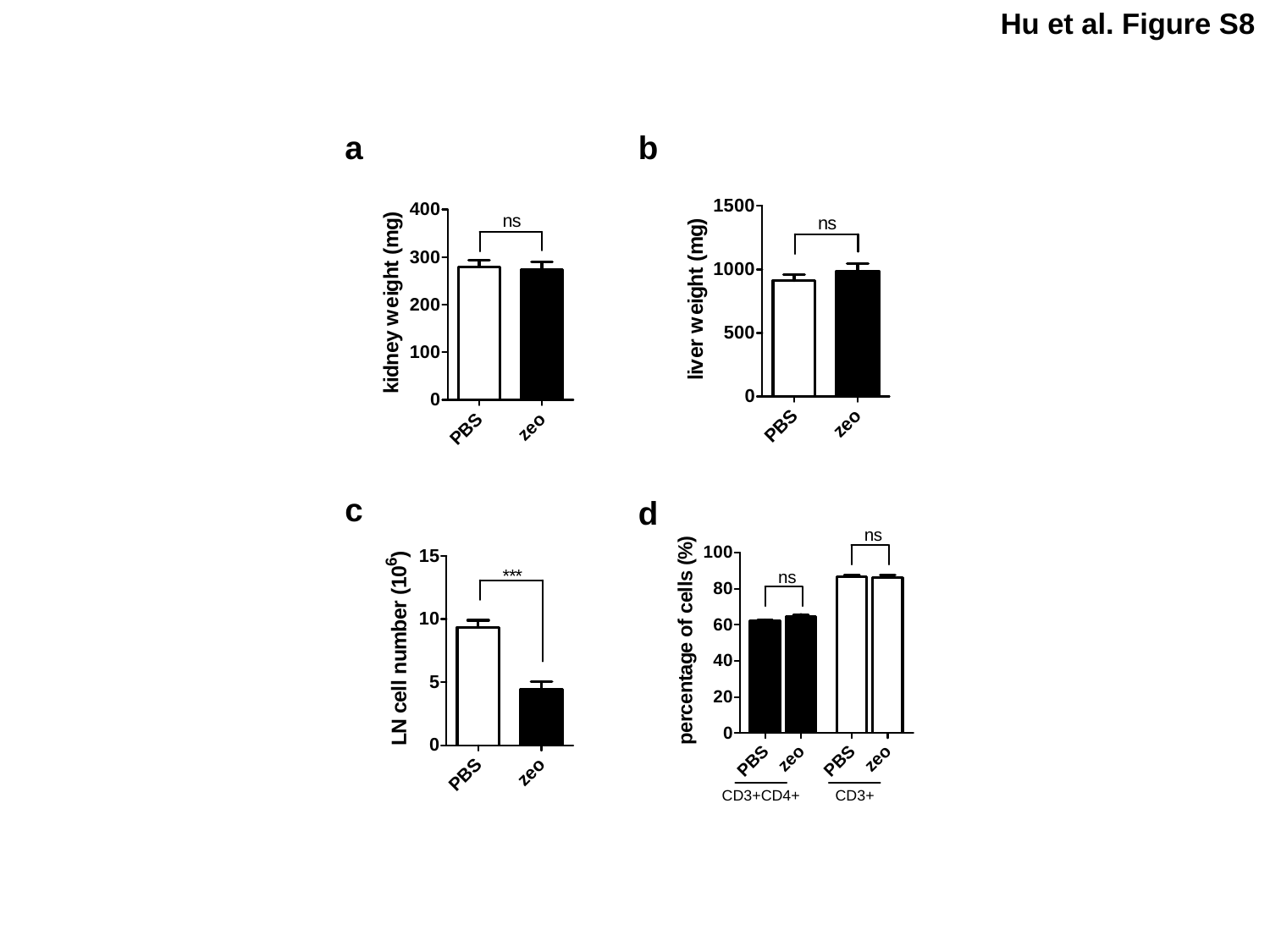

Hu et al. Figure S8
a
b
c
d
