# Supplementary material for: Resting T cells are hypersensitive to DNA damage due to defective DNA repair pathway
Source: Cell Death Dis. 2018 May 31;9(6):662. doi: 10.1038/s41419-018-0649-z (PMC5981309; doi:10.1038/s41419-018-0649-z)
Supplement: Supplementary file 2 — Supplemetary Figure legend [file 41419_2018_649_MOESM2_ESM.docx]

**Supplementary Figure 1.** Related to Figure 1. (**a**) Freshly isolated (resting) human CD4+ T cells were treated with PBS and released for the indicated time. The percentage of apoptotic (Annexin V positive) cells from each time points were then analyzed by flow cytometry. Ctl indicates resting T cells without treatment. All values are the average ± SEM of three independent experiments.

**Supplementary Figure 2.** Related to Figure 1. Resting (**a**) and stimulated (**b**) CD4+ T cells were irradiated with 2 Gy X-ray and released for the indicated time. Apoptotic cells were analyzed by flow cytometry. Ctl indicates resting or stimulated T cells without irradiation. (**c**) Quantitation of the percentage of apoptotic (Annexin V positive) cells in (a) and (b). (**d**) The level of cleaved PARP in T cells were detected by western blot. (**e**) Resting and stimulated CD4+ T cells were treated with 25 μM H_2_O_2_ for 10 min and released for the indicated time. The percentage of apoptotic (Annexin V positive) cells from each time points were then analyzed by flow cytometry. Ctl indicates resting or stimulated T cells without H_2_O_2_ treatment. All values are the average ± SEM of three independent experiments. The unpaired student’s two-tailed t-test was used to determine the statistical significance (* P<0.05, ** P<0.01, *** P<0.001).

**Supplementary Figure 3.** Related to Figure 2. (**a**) Representative results of alkaline comet assay. Resting or stimulated CD4+ T cells were treated with 25 μM H_2_O_2_ for 10 min then released for the indicated times. Ctl indicates resting or stimulated T cells without H_2_O_2_ treatment. (**b** and **c**) The percentage of DNA in the tail for each resting CD4+ T cell (b) and the percentage of resting T cells with over 10% tail DNA (c) were measured. Cells were isolated from 3 healthy donors, including control (n=323), 0 h after zeocin treatment (n=160) and 4 h after zeocin treatment (n=305). (**d** and **e**) The percentage of DNA in the tail for each stimulated CD4+ T cell (d) and the percentage of stimulated cells with over 10% tail DNA (e) were measured, including control (n=313), 0 h after zeocin treatment (n=198) and 4 h after zeocin treatment (n=245). All values are the average ± SEM of three independent experiments. The unpaired student’s two-tailed t-test was used to determine the statistical significance (* P<0.05, ** P<0.01, *** P<0.001).

**Supplementary Figure 4.** Related to Figure 3. (**a**) Resting and stimulated CD4+ T cells were irradiated with 2 Gy X-ray and released for time. Untreated (non-damaged) resting and stimulated T cells were used as negative control (ctl). γH_2_AX foci were analyzed by immunofluorescence. Cell nuclei were stained with DAPI as shown in the “merge” images. (**b**) Quantitation of the data in (a). The average number of γH_2_AX foci per cell was determined. (**c**) Resting and stimulated CD4+ T cells were irradiated with 2 Gy X-ray and released for indicated times. 53BP1 foci were analyzed by immunofluorescence. (**d**) Quantitation of the data in (c). The average number of 53BP1 foci per cell was determined. All values are the average ± SEM of three independent experiments.

**Supplementary Figure 5.** Related to Figure 3. Resting and stimulated CD4+ T cells from each time point post zeocin treatment were analyzed by Q-PCR. Ctl indicates resting or stimulated T cells without zeocin treatment. (**a**) Q-PCR analysis of NHEJ-related proteins. (**b**) Q-PCR analysis of HR-related proteins. (**c**) Q-PCR analysis of Fanconi Anaemia pathway-related proteins. (**d**) Q-PCR analysis of CHK1 and CHK2. All values are the average ± SEM of three independent experiments.

**Supplementary Figure 6.** Related to Figure 3. Resting and stimulated CD4+ T cells were treated with 200 μg/ml zeocin for 1 h and released at the indicated time. Ctl indicates resting or stimulated T cells without zeocin treatment. The phosphorylation of DNA-PKcs (**a**) and H_2_AX (**b**) were analyzed by western blot.

**Supplementary Figure 7.** Related to Figure 5. (**a**) The resting T cells from p53 knockout mice were treated with 200 μg/ml zeocin for 1 h or 25 μM H_2_O_2_ for 10 min and then released for 1 d. The percentage of apoptotic (Annexin V positive) cells were analyzed by flow cytometry. Data are representative of resting T cells from four mice for each group. All values are the average ± SEM. (**b**) Q-PCR analysis of NOXA and PTEN in human resting and stimulated CD4+ T cells from each time point post zeocin treatment. All values are the average ± SEM of three independent experiments.

**Supplementary Figure 8.** Related to Figure 6. Six-weeks-old female BALB/c mice were injected with PBS or zeocin (320 mg/kg) and sacrificed three days later. (**a**) The total weights of two kidneys from mice treated with PBS or zeocin were determined. (**b**) The weights of livers from mice treated with PBS or zeocin were determined. (**c**) The total cell number in the two subiliac lymph nodes (LN) from mice treated with PBS or zeocin was counted. (**d**) Quantitation of the proportion of T cell (CD3+) or CD4+ T cell (CD3+CD4+) in subiliac lymph node cells. Data are representative of five to six mice for each group. All values are the average ± SEM. The unpaired student’s two-tailed t-test was used to determine the statistical significance (* P<0.05, ** P<0.01, *** P<0.001).
