# Supplementary material for: Resting T cells are hypersensitive to DNA damage due to defective DNA repair pathway
Source: Cell Death Dis. 2018 May 31;9(6):662. doi: 10.1038/s41419-018-0649-z (PMC5981309; doi:10.1038/s41419-018-0649-z)
Supplement: Supplementary file 3 — Supplementary table [file 41419_2018_649_MOESM3_ESM.docx]

**Supplementary Table:**

Primers used in Q-PCR:

| Primers | Sequences |
| --- | --- |
| CD3E-forward primer | 5’-GGCAGGCAAAGGGGACA-3’ |
| CD3E-reverse primer | 5’-ACCATGAGGCTGAGGAACGAT-3’ |
| MKP1-forward primer | 5’-AGTACCCCACTCTACGATCAGG-3’ |
| MKP1-reverse primer | 5’-GAAGCGTGATACGCACTGC-3’ |
| FASL-forward primer | 5’-TGCCTTGGTAGGATTGGGC-3’ |
| FASL-reverse primer | 5’-GCTGGTAGACTCTCGGAGTTC-3’ |
| FASR-forward primer | 5’-TCTGGTTCTTACGTCTGTTGC-3’ |
| FASR-reverse primer | 5’-CTGTGCAGTCCCTAGCTTTCC-3’ |
| PUMA-forward primer | 5’-GACCTCAACGCACAGTACGAG-3’ |
| PUMA-reverse primer | 5’-AGGAGTCCCATGATGAGATTGT-3’ |
| BCL-2-forward primer | 5’-GGTGGGGTCATGTGTGTGG-3’ |
| BCL-2-reverse primer | 5’-CGGTTCAGGTACTCAGTCATCC-3’ |
| NOXA-forward primer | 5’-ACCAAGCCGGATTTGCGATT-3’ |
| NOXA-reverse primer | 5’-ACTTGCACTTGTTCCTCGTGG-3’ |
| BAX-forward primer | 5’-CCCGAGAGGTCTTTTTCCGAG-3’ |
| BAX-reverse primer | 5’-CCAGCCCATGATGGTTCTGAT-3’ |
| BIM-forward primer | 5’-TAAGTTCTGAGTGTGACCGAGA-3’ |
| BIM-reverse primer | 5’-GCTCTGTCTGTAGGGAGGTAGG-3’ |
| PTEN-forward primer | 5’-TGGATTCGACTTAGACTTGACCT-3’ |
| PTEN-reverse primer | 5’-GGTGGGTTATGGTCTTCAAAAGG-3’ |
| TAp73-forward primer | 5’-TGGAACCAGACAGCACCTAC-3’ |
| TAp73-reverse primer | 5’-CTGGGCCATGACAGATGTAG-3’ |
| KU70-forward primer | 5’-GTTCTAAAGGTCTTTGCAGCAAGA-3’ |
| KU70-reverse primer | 5’-AAAAGCCACGCCGACTTGAGGA-3’ |
| KU80-forward primer | 5’-GGTTTCAAGCCGTTGGTACTGC-3’ |
| KU80-reverse primer | 5’-CTCCAGACACTTGATGAGCAGAG-3’ |
| LIG4-forward primer | 5’-CAGCAGAGATCGTACCCAGTGA-3’ |
| LIG4-reverse primer | 5’-TGCGAGCTTACCAGATGCCTTC-3’ |
| XRCC4-forward primer | 5’-ATGGCTCCTCAGGAGAATCAGC-3’ |
| XRCC4-reverse primer | 5’-GAGGTCTTCTGGGCTGCTGTTT-3’ |
| BRCA1-forward primer | 5’-CTGAAGACTGCTCAGGGCTATC-3’ |
| BRCA1-reverse primer | 5’-AGGGTAGCTGTTAGAAGGCTGG-3’ |
| BRCA2-forward primer | 5’-GGCTTCAAAAAGCACTCCAGATG-3’ |
| BRCA2-reverse primer | 5’-GGATTCTGTATCTCTTGACGTTCC-3’ |
| RAD51- forward primer | 5’-TCTCTGGCAGTGATGTCCTGGA-3’ |
| RAD51-reverse primer | 5’-TAAAGGGCGGTGGCACTGTCTA-3’ |
| FANCA- forward primer | 5’-CTCAAGGGTCAGGGCAACC-3’ |
| FANCA- reverse primer | 5’-TTTCGGGCACCGAGGTATTA-3’ |
| FANCD2- forward primer | 5’-CATGGCTGTTCGAGACTTCA-3’ |
| FANCD2- reverse primer | 5’-TGATGAAGCAGCCTTGTGTC-3’ |
| FANCE- forward primer | 5’-CCTATGCCAAGCTCATGCTGA-3’ |
| FANCE- reverse primer | 5’-CCAAATGTTTCAAGGCGGC-3’ |
